# Affective bodily responses in monkeys predict subsequent pessimism, but not vice versa

**DOI:** 10.1098/rspb.2024.2549

**Published:** 2025-02-05

**Authors:** Sakumi Iki, Ikuma Adachi

**Affiliations:** ^1^ Center for the Evolutionary Origins of Human Behavior, Kyoto University, Inuyama, Aichi, Japan

**Keywords:** behavioural contagion, cognitive bias, dual-process theory, judgement bias, peripheral origin theory, self-directed behaviour

## Abstract

Affect is a complex interplay of behaviour, physiology, cognition and subjective feelings. The James–Lange theory, which posits that affective bodily responses occur first and subsequently shape our affective experiences, has been extensively studied in humans but remains underexplored in non-human animals. This study employs a judgement bias test to explore the relationship between peripheral bodily responses, specifically self-scratching associated with negative affect, and shifts in cognitive information-processing modes (i.e. a ‘pessimistic’ judgement bias characterized by heightened anticipation of undesirable outcomes) in Japanese macaques (*Macaca fuscata*). Our findings support the hypothesis that bodily responses precede and influence changes in cognitive modes, demonstrating that self-scratching predicts subsequent pessimistic judgements, but not vice versa. This study provides comparative insights into the biological underpinnings of affect, highlighting that peripheral behaviours can shape cognitive processes in non-human primates. These results have broader implications for understanding the evolutionary continuity and adaptive significance of affective response systems across species.

## 1. Background

Affect is a multifaceted phenomenon, manifesting as an intertwined combination of several aspects, such as behaviour, physiology, cognition and subjective feelings [1–3]. The interplay among these aspects has been a subject of intense discussion in biological, psychological and philosophical realms [4–7]. A classic hypothesis concerning the relationship between aspects of affect that has attracted the attention of both the academic community and the general public is the James–Lange theory [5,6,8]. This hypothesis specifically addresses the relationship between affective peripheral (i.e. bodily) changes and the subjective experience of affect, proposing that affective bodily responses occur first and subsequently shape our subjective affective experiences [9,10]. William James’s famous phrase—‘we feel sorry because we cry, angry because we strike, afraid because we tremble,’ rather than ‘we cry, strike, or tremble, because we are sorry, angry, or fearful’—well illustrates this view [6, p. 190].

The James–Lange hypothesis has stimulated numerous studies and led to refinements in understanding the relationship between peripheral bodily changes triggered by emotionally evocative stimuli and central cognitive processing (e.g. [11–15]). However, because the James–Lange hypothesis emphasises subjective affective experience [9,10], empirical investigations into the interrelation between affective peripheral/bodily responses (e.g. behaviour, physiology) and central/cognitive processing (e.g. judgement, decision-making, reasoning) have predominantly centred on human subjects, who can linguistically articulate their personal experiences through introspection [16,17]. Consequently, despite the wealth of data on the biological foundation of the affective system derived from rigorous neuroscientific studies on animal models (e.g. [17]), research from a comparative perspective on this topic in non-human animals remains relatively scarce.

**Figure 1.**
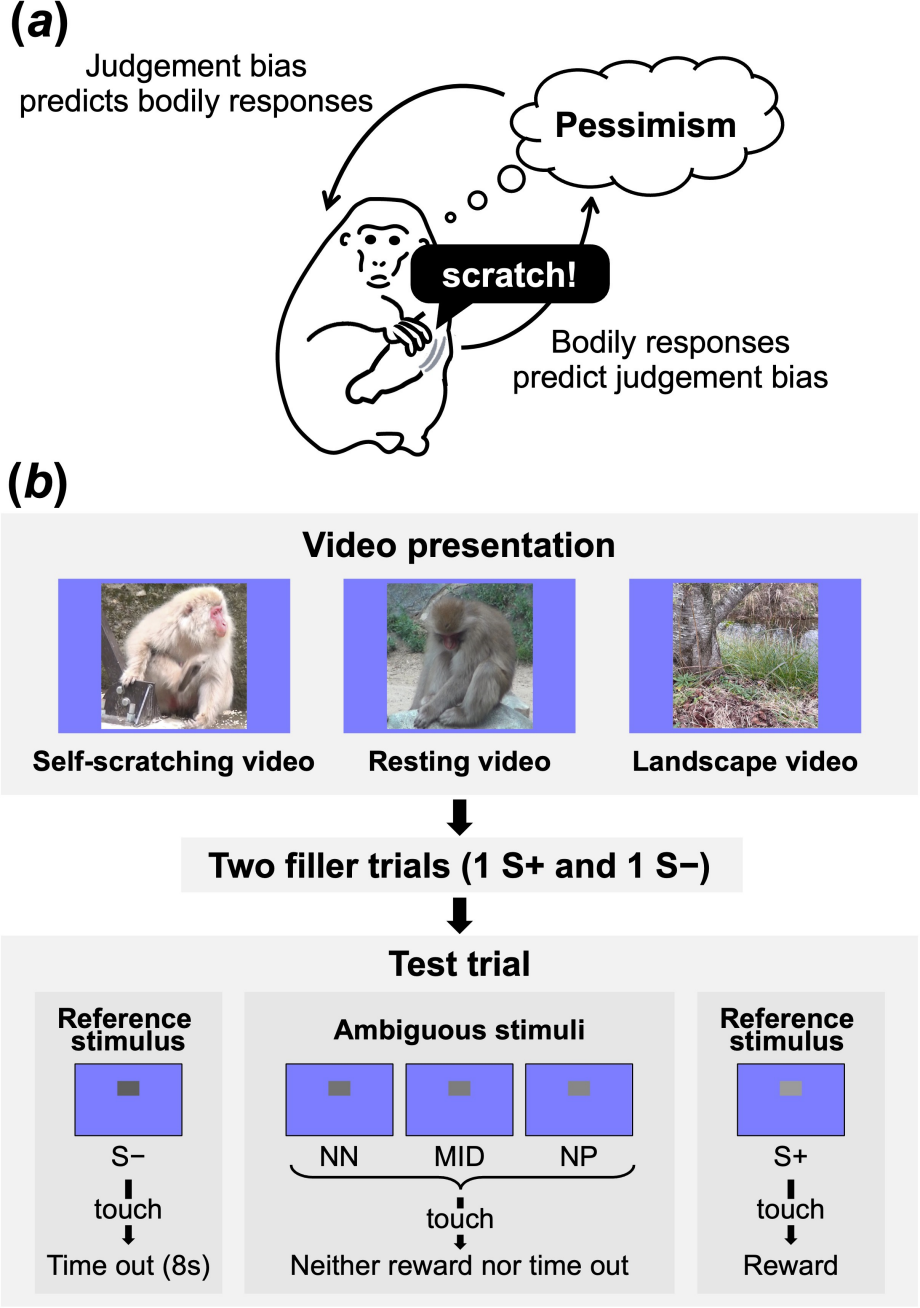
Overview of the hypotheses and experimental protocol. (*a*) This study tested whether subjects’ preceding self-scratching predicts subsequent pessimistic judgements (i.e. lowered expectations of favourable outcomes) in the judgement bias test, and whether preceding pessimistic judgements in the judgement bias test predict subsequent occurrences of self-scratching. (*b*) Each session consisted of 20 judgement bias test trials, each preceded by a 3 s video presentation. Between the video presentation and the test trial, two filler trials of a go/no-go visual discrimination task were conducted. We adopted a block design, exclusively using either conspecific scratching videos, conspecific resting videos or landscape videos within a single session. During each test trial, a single greyscale stimulus was presented, selected from five types of test stimuli: two trained reference stimuli (S+ and S−) and three ambiguous stimuli (near positive (NP), middle (MID) and near negative (NN)). Each of the five greyscale test stimuli was used four times per session in a counterbalanced and pseudorandomized order. The allocation of dark and light tones to S+ and S– was also counterbalanced across subjects.

Recent advances in experimental methodologies may offer fresh insights into the enduring theme of the interplay between central (cognitive) and peripheral (bodily) facets of affect by allowing us to work with non-human animals, which lack the capacity for verbal reports of their experiences. Affective states are accompanied by specific cognitive information-processing modes, as described below, that align with their specific valence [18,19]: during negative affect, mental processing slows down, and judgements become more ‘pessimistic’ (i.e. heightened anticipation of undesirable outcomes), whereas positive affect induces the opposite cognitive changes. The judgement bias test is a method to estimate the affective state of animals by measuring whether they make pessimistic or optimistic judgements in ambiguous situations [20,21]. This method has been used across diverse disciplines, encompassing animal psychology, animal welfare and pharmacology, and its validity has been corroborated across a range of taxa, including invertebrates, fishes, birds and mammals (reviewed in [21–23]). Using this method, this study aims to examine the relationship between peripheral bodily responses linked with negative affect and the pessimistic cognitive mode in non-human primates (Japanese macaques, *Macaca fuscata*) (figure 1*a*).

To address the aforementioned issue, we focused specifically on self-scratching behaviour, a somatomotor response associated with negative affect. Our rationale for targeting this behaviour is threefold. First, the association between self-scratching and negative affect has been robustly validated by pharmacological and physiological studies using anxiogenic and anxiolytic drugs [24,25]. Second, self-scratching behaviour is prevalent in non-human primates in situations associated with negative affect such as anxiety and fear (Japanese macaques: [26–28]; long-tailed macaques, *Macaca fascicularis*: [24]; olive baboons, *Papio anubis*: [29]; chimpanzees, *Pan troglodytes*: [30]; brown lemurs, *Eulemur fulvus*: [31]). Therefore, focusing on this behaviour facilitates discussion from a comparative perspective. Third, several studies have suggested that self-scratching behaviour exhibits contagion among individuals and can be experimentally induced (humans, *Homo sapiens*: [32]; mice, *Mus musculus*: [33]; rhesus macaques, *Macaca mulatta*: [34]; Bornean orangutan, *Pongo pygmaeus*: [35]; Geoffroy’s spider monkeys, *Ateles geoffroyi*: [36]; but see [37,38]). Building on this third point, we aimed to induce self-scratching in our subjects by presenting videos of conspecifics engaging in self-scratching. We then examined the relationship between subjects’ self-scratching and judgement bias, and tested whether the former predicts the latter, vice versa, or if they bi-directionally predict each other.

This study adopted a within-subject design, where six captive Japanese macaques were subjected to a touchscreen-based go/no-go judgement bias test (figure 1*b*; see §2 for details). The subjects performed go/no-go visual discrimination tasks requiring them to distinguish between dark- and light-grey stimuli predictive of a reward (i.e. a food pellet) or a mild punishment (i.e. an 8000 ms time out). These tasks were interspersed with video presentations under three conditions (conspecific self-scratching video, conspecific resting video and landscape video; see §2). After each video presentation, two filler discrimination trials were administered, followed by a judgement bias test trial in which an ambiguous intermediate-grey stimulus was presented. Subjects exhibiting a pessimistic judgement bias are expected to show hesitation in interpreting an ambiguous stimulus as a rewarding stimulus, resulting in slower ‘go’ responses to the ambiguous stimulus. By recording the timing of subjects’ self-scratching during the experimental sessions, we addressed the following key questions: (i) whether subjects’ preceding self-scratching predicted subsequent pessimistic judgements in the judgement bias trial, and (ii) whether preceding pessimistic judgements in the last judgement bias trial predicted subsequent occurrences of self-scratching.

Specifically, we tested the following three mutually exclusive hypotheses. The first posits that bodily responses (i.e. self-scratching) would instigate afferent changes, resulting in a cognitive mode associated with negative affect (i.e. pessimistic judgement bias). This hypothesis aligns with the facial feedback phenomenon reported in humans [39,40], in which subjects tend to evaluate an object favourably (or unfavourably) when induced to make positive (or negative) facial expressions. We denote this hypothesis as the ‘*BR→CM hypothesis*’ (BR signifies ‘bodily responses,’ and CM represents ‘cognitive mode’). Under this hypothesis, we expected that following self-scratching, subjects would exhibit diminished expectations of favourable outcomes when confronted with ambiguous situations. Consequently, we predicted that when the judgement bias test trial was preceded by the subjects’ self-scratching, it would result in an increased response time to the ambiguous stimuli. We also predicted that pessimistic judgements by the subjects would not necessarily be followed by self-scratching.

The second alternative hypothesis posits that the pessimistic cognitive mode instigates efferent changes, resulting in a specific bodily response. We denote this hypothesis as the ‘*CM→BR hypothesis*.’ Under this hypothesis, we predicted that the response time to the ambiguous stimuli would not necessarily increase when the judgement bias test trial was preceded by the subjects’ self-scratching. Meanwhile, we predicted that pessimistic judgements by the subject (i.e. increased response time to ambiguous stimuli) would result in the increased occurrence of subsequent self-scratching.

The third hypothesis posits that the bodily response and the change in cognitive mode form a feedback loop. We denote this hypothesis as the ‘*BR⇄CM hypothesis*.’ This hypothesis aligns with findings from human studies (e.g. [14,41]), suggesting that bodily responses to emotionally evocative stimuli instigate central/cognitive alterations, which in turn amplify the former. Under this hypothesis, we predicted that the response time to the ambiguous stimuli would increase when the judgement bias test trial was preceded by the subjects’ self-scratching, and that the subjects’ pessimistic judgement (i.e. the prolonged response time to the ambiguous stimuli) would result in an increased occurrence of subsequent self-scratching. The predictions derived from each of the above three hypotheses are summarized in table 1.

**Table 1.** Overview of the hypotheses and predictions. (BR, bodily responses; CM, cognitive modes.)

hypothesis	prediction 1: preceding self-scratching predicts subsequent pessimistic judgement	prediction 2: preceding pessimistic judgement predicts subsequent self-scratching
BR→CM	yes	no
CM→BR	no	yes
BR⇄CM	yes	yes

Furthermore, although it does not directly pertain to our key questions, given that previous studies have indicated that scratching is contagious among conspecifics across various animal species, we predicted that subjects would be more likely to scratch themselves in the scratching video condition compared with the resting and landscape video conditions. Moreover, given evidence linking self-scratching to negative affect [26–28], viewing a conspecific’s self-scratching may induce negative emotional contagion in the subjects. Consequently, we also predicted that subjects would make more pessimistic judgements in the scratching video condition than in the other two conditions.

## 2. Methods

### (a) Study subjects

We conducted tests on six adult Japanese macaques at the Center for the Evolutionary Origins of Human Behavior (EHUB), Kyoto University. These macaques comprised two males and four females aged 8 to 18 years (13.00 ± s.d. 3.52 years old; see the electronic supplementary material, table S1). Four of the subjects (two males, two females) had received equivalent go/no-go discrimination training for a different study more than five months before this study, while the remaining two had no prior experience with such tasks. The subjects were housed either individually or in pairs. In the case of paired housing, a partition was introduced at least 30 min prior to the experiment to separate them. To control the subjects’ motivation for reward, they were not fed for approximately 20 h prior to the trial. Because our subjects are accustomed to a 24 h feeding interval on weekends, we consider this fasting period unlikely to induce any intense negative affect. Upon completion of the day’s experiment, the experimenter fed the subjects daily rations of 450 g of monkey chow (AS, Oriental Yeast Co., Ltd.), supplemented with radish and carrot three times a week. Water was made available to the subjects ad libitum.

### (b) Experimental paradigm

#### (i) Video stimuli

The test sessions included three conditions: the scratching video condition, the resting video condition (i.e. social control condition) and the landscape video condition (i.e. non-social control condition). We used 165 video stimuli in our experiments, each with a duration of 3 s and devoid of sound. These stimuli included 55 videos each of conspecific scratching, conspecific resting and landscapes. Scratching and resting videos were recorded using a digital video camera (HDR-TD10; Sony Corporation, Tokyo, Japan) at Jigokudani Monkey Park (Nagano Prefecture, Japan). Landscape videos were gathered from the vicinity of the EHUB. Unlike yawning, which has been demonstrated in multiple species to be more contagious among individuals with stronger social bonds or familiarity (e.g. humans and bonobos, *Pan paniscus*: [42]; dogs, *Canis lupus familiaris*: [43]; wolves, *Canis lupus lupus*: [44]; but see [45] for a study showing that yawning is more contagious among weakly bonded individuals), a study investigating the social bias of scratching contagion discovered a significantly higher likelihood of contagion among individuals with weaker social bonds [35]. In light of this finding, we employed videos featuring unfamiliar individuals, anticipating that they would elicit stronger contagion. Scratching was defined as repeated raking of the surface of one’s own body with one’s fingers or toes [37], while resting was defined as the state where an individual maintains a sitting or lying position without any apparent behavioural manifestation. The scratching and resting videos were required to satisfy the following criteria: each video displayed only one individual with nearly the entire body visible to the camera; the individual featured in the video was an adult aged over 4 years; the individual in the video was not engaging in direct gaze with the camera (as direct gaze is considered a threat signal in Japanese macaques) and was not exhibiting any explicit communicative signals; and, in the case of scratching, the body part being scratched was visible to the camera. Landscape videos were chosen for experimental use if they featured natural objects (e.g. trees, grass, rivers) and did not include any animals. Among the 55 scratching and resting videos, five from each category featured males. This sex bias was owing to the generally lower number of adult males compared with adult females in Japanese macaque groups, which makes the collection of self-scratching videos of adult males relatively challenging.

#### (ii) Apparatus

The reference and ambiguous greyscale stimuli (300 × 200 pixels) and the video stimuli (900 × 900 pixels) were displayed via a touchscreen (Microsoft Surface Pro 8 EIV-00010). The touchscreen was placed in front of each subject’s cage, with no mesh or bars between the screen and the subject, ensuring an unobstructed direct view of the screen. Upon a correct response, a food dispenser (Med Associates, Fairfax, Vermont, USA) automatically released a banana-flavoured pellet (Bio-Serv DPP 190 mg) from the tray located beneath the screen, accompanied by a 1 s beep. An incorrect response from the subject would trigger a buzzing signal. Visual Basic 2010 (Microsoft Corporation, Redmond, Washington, USA) was employed to control the experimental apparatus. During the experiment, we recorded the timing of each subject’s self-scratching using GoPro Hero11 video cameras, positioned on top of the touchscreen and above the cage. To synchronize the video data with the computer-based trial timeline, we used the onset of a beep emitted at the start of the experimental session as a reference point. A scratching instance that occurred more than 3 s following the conclusion of the previous scratching was classified as a new bout.

#### (iii) Training phase

The training and testing protocols adhered to the study conducted by Iki & Adachi [46] on the same species. The subjects underwent training in a go/no-go visual discrimination task from June 2023 to January 2024. Three dark grey (red/green/blue (RGB) 75/75/75, 85/85/85 and 95/95/95) and three light grey stimuli (RGB 155/155/155, 165/165/165 and 175/175/175) served as reference stimuli, acting as predictors of a reward pellet (S+) and mild, non-invasive punishment (i.e. an 8000 ms time out) (S−). To prevent subjects from habitually approaching a single point on the greyscale, three distinct grey tones with slight variations in brightness were allocated to S+ and S−, respectively. The allocation of dark and light tones to S+ and S− was counterbalanced between subjects. S+ and S− stimuli were displayed in the upper centre of the screen for 2 s after the subjects touched the self-start key located in the lower centre of the screen. The order of S+ and S− stimuli was pseudorandomized, with all six reference stimuli appearing every six trials. S+ was always presented at the very beginning of each training session. Upon a subject’s touch on S+, the stimulus disappeared, and a reward pellet was dispensed. An 80% variable reinforcement ratio was introduced to lessen the learning effects of unrewarding ambiguous stimuli [47,48]. Specifically, in 80% of S+ trials, once the pellet was dispensed, the inter-trial interval (ITI; a 2000 ms blank screen in light blue) began. In the remaining 20% of S+ trials, the ITI began immediately after the subject touched the S+, without dispensing any reward. At the end of the ITI, the self-start key reappeared. In response to a touch on S−, the stimulus disappeared, and the subject was required to wait an 8000 ms time out in addition to the ITI prior to advancing to the subsequent trial.

The training protocol was composed of two distinct stages. In the initial stage, subjects performed as many S+ and S− discrimination trials as possible within 1 h. Before the start of the session, the touchscreen display was blacked out. After a 90 s interval following the experimenter’s keyboard operations, the screen transitioned to a start screen with a green background for a duration of 2 min. The experimenter left the experimental room during the 90 s interval; after 2 min, the background changed to a light blue colour, the self-start key was displayed, and the training session began. Subsequent to each day’s training, we calculated the proportions of correct go and no-go responses. Subjects who completed a minimum of 100 training trials daily and whose proportions of correct go and no-go responses were 0.9 or higher for a minimum of three consecutive days advanced to the subsequent training stage.

The subsequent training stage was designed to familiarize subjects with the presentation of videos, given that mere exposure to videos, even in the absence of emotionally evocative content, could potentially provoke adverse affective responses in subjects unaccustomed to encountering video stimuli on a touchscreen. For this training stage, we collected 20 landscape videos, each 3 s in duration, which were distinct from the landscape videos used in the testing phase. Following the same procedure as in the initial training stage, subsequent to the display of the 2 min green start screen, the background transitioned to a light blue colour, the self-start key was exhibited and the training session was started. Training at this stage proceeded as follows:

1. forty trials of the discrimination task were executed, with S+ and S− each presented an equal number of times;
2. ten trials of the discrimination task were executed, with S+ and S− each presented an equal number of times, followed by the presentation of the same self-start key used in the go/no-go trials. When the subject touched the self-start key, a landscape video was displayed. A 500 ms interval was interposed between the termination of the video presentation and the onset of the self-start key presentation;
3. the procedure described in step 2 was reiterated 20 times. The video was pseudorandomly chosen from a dataset comprising 20 videos; and
4. ten trials of the discrimination task were conducted, with S+ and S− each presented an equal number of times.

Each training session thus consisted of 250 go/no-go trials in total. Subjects who completed all steps 1 through to 4 and whose proportions of correct go and no-go responses were 0.9 or greater for a minimum of three consecutive days were eligible to advance to the testing phase. All six subjects, including those without prior experience in go/no-go visual discrimination tasks, met the learning criteria.

#### (iv) Testing phase

Experimental testing was carried out from January to February 2024. Two dark grey (RGB 85/85/85 and 95/95/95) and two light grey stimuli (RGB 155/155/155 and 165/165/165) were used as S+ and S−. To increase the difficulty in differentiating between the ambiguous and reference stimuli, reference stimuli most distant from the ambiguous stimuli (RGB 75/75/75 and 175/175/175) were excluded from the testing. Ambiguous stimuli consisted of three intermediate grey stimuli (RGB 115/115/115, 125/125/125 and 135/135/135), which were designated as near positive (NP), middle (MID) and near negative (NN), contingent on their proximity to the S+ and S− stimuli. Testing was conducted on weekdays, employing a block design experiment that alternated daily between scratching video, resting video and landscape video conditions, with each condition comprising five sessions. The order of the conditions was counterbalanced across subjects. The 55 scratching videos were divided into five subsets of 11 videos each, as were the 55 resting and 55 landscape videos. Given that we conducted five sessions for each of the scratching, resting and landscape conditions, the five subsets of videos for each condition were used solely once for each individual. The order of the subset of videos was determined pseudorandomly. Each test session encompassed 21 instances of video presentations, with one of the 11 videos from each subset used solely once in the initial video presentation of a session, and the residual 10 videos used twice. Following the same procedure as in the training phase, subsequent to the display of the 2 min green start screen, the background transitioned to a light blue colour, the self-start key was exhibited and the testing session was started. The detailed procedure of the testing phase was as follows:

1. forty filler trials of the discrimination task were conducted, with S+ and S− each presented an equal number of times. Upon completion of the 40 trials, the proportions of correct go and no-go responses were calculated. If a subject’s proportion of correct responses was 0.9 or greater, they advanced to the subsequent step. If the proportion was less than 0.9, an additional 150 trials of the discrimination task were administered, and the subject did not advance to the subsequent steps for that day;
2. ten filler trials of the discrimination task were conducted, followed by the presentation of the same self-start key used in the go/no-go trials. When the subject touched the self-start key, a scratching, resting or landscape video was displayed, contingent on the condition of the day. A 500 ms interval was interposed between the termination of the video presentation and the self-start key presentation;
3. three filler trials of the discrimination task were conducted, comprising two trials of S+ and one trial of S− (RGB 95/95/95 and 155/155/155). S+ and S− were exhibited in a sequence counterbalanced within and across subjects, with the third trial invariably showcasing S+;
4. ten filler trials of the discrimination task were conducted, where the reference stimuli with RGB values of 85/85/85 and 165/165/165 were exhibited three times each, and 95/95/95 and 155/155/155 were displayed twice each, presented in a pseudorandom order;
5. when the subject touched the self-start key, a scratching, resting or landscape video was exhibited, contingent on the condition of the day. A 500 ms interval was interposed between the termination of the video presentation and the onset of the self-start key presentation;
6. two filler trials of the discrimination task were conducted, each comprising one trial of S+ and one trial of S− (RGB 95/95/95 and 155/155/155), presented in a sequence counterbalanced within and across subjects. These two filler trials were introduced to enable the subjects to ascertain that touching the self-start key would result in the presentation of the greyscale stimuli, rather than the presentation of videos;
7. a single test trial was executed, during which one of the following five test stimuli was presented for 2000 ms: S+, S− (RGB 95/95/95 and 155/155/155), or one of the three types of ambiguous stimuli. If subjects touched the ambiguous stimulus, it disappeared; the ambiguous stimulus did not predict either a reward or mild punishment. As an index of the judgement bias, the response time to the test stimulus was recorded. Following previous studies [47–50], and to integrate no-go responses into this index, we assigned a ceiling latency of 2000 ms (i.e. the maximum presentation duration) as the response time whenever the subject did not touch the test stimulus;
8. steps from 4 to 7 were reiterated 20 times. Given that we adopted a block design experiment, we solely presented either 20 scratching videos, 20 resting videos or 20 landscape videos within a single session. Each of the five greyscale test stimuli was exhibited four times in each session. The order of the test stimuli was counterbalanced within and across subjects. The combination of the test stimuli and the preceding videos was also counterbalanced within and across subjects; and
9. ten filler trials of the discrimination task were conducted, with S+ and S− each presented an equal number of times.

Each test session consisted of 323 go/no-go trials in total. An 80% variable reinforcement ratio was applied in steps 1, 2, 4 and 9. Throughout the span of 15 sessions, each of the five test stimuli was exhibited to each subject 20 times in all of the three conditions, culminating in a total of 1800 ( = 5 test stimuli (S+, S−, NP, MID, NN) × 4 presentations per session × 5 sessions per condition × 3 conditions (scratching, resting, landscape) × 6 subjects) recorded response times to the test stimuli. Because no prior research has examined the relationship between self-scratching and judgement bias, we lacked any prior knowledge of the expected effect size and therefore did not conduct a power analysis. Following a previous study [46] that employed a similar touchscreen-based judgement bias task in Japanese macaques, we presented each type of test stimulus 20 times per condition.

### (c) Statistics

We analysed the data using generalized linear mixed models (GLMMs) with the glmer function in the lme4 package in R version 4.1.2. We set our alpha level to 0.05.

Regarding the response time to the test stimuli, we employed a GLMM with a gamma error structure and a log link function. As key predictors, we included the type of test stimulus (categorical: S+, S−, NP, MID, NN), the number of bouts of self-scratches occurring during the sub-block between the conclusion of the last judgement bias test trial and the beginning of the current judgement bias test trial (continuous), and a two-way interaction between these factors. We also included the condition (categorical: landscape, resting, scratching). To control for potential confounding effects, we included the following control variables: session number (continuous: day 1−15), trial number (continuous: 1−20), subject’s sex (categorical), sex of the individual in the video stimulus (categorical) and baseline frequency of self-scratching (continuous). Two-way interactions between these factors and the type of test stimulus were also included. The sex of the individual in the video stimulus was coded as ‘1’ when the individual was male, and ‘0’ in all other cases (including the landscape condition). This coding was used because males are dominant in Japanese macaques and can, therefore, pose a greater threat to individuals. The baseline frequency of self-scratching was calculated as the number of self-scratches occurring during the 2 min green start screen period. To address pseudoreplication, we included subject identity (ID) as a random intercept and a random slope for the type of test stimuli. Given that our analytical objective was hypothesis testing and that evidence indicates stepwise model reduction is unsuitable for such purposes—because it can yield biased coefficients and inflate Type I error rates [51,52]—we did not employ such model reduction methods. Instead, we conducted a simultaneous significance test of all predictor variables using the full model, a method considered more desirable for hypothesis testing [51,52]. The significance of the full model was confirmed through comparison with a null model containing control variables and a random intercept, using a likelihood ratio test with the anova function (electronic supplementary material, table S2).

For the occurrence of self-scratching in the sub-block between the end of the last judgement bias test trial and the onset of the current judgement bias test trial, we employed a GLMM with a Poisson error structure and a log link function. The response variable was the number of bouts of self-scratches occurring during the sub-block. Key predictors included the type of test stimulus in the last judgement bias test trial (categorical: S+, S−, NP, MID, NN; for the first test trial, we assigned the S+ from the third trial in step 3 of §2b(iv) above), the response time to the test stimulus in the last judgement bias test trial (continuous), and a two-way interaction between these factors. The condition (categorical: landscape, resting, scratching) was also included. To control for potential confounding effects, we included the following control variables: session number (continuous: day 1−15), trial number (continuous: 1−20), subject’s sex (categorical), sex of the individual in the video stimulus (categorical), baseline frequency of self-scratching (continuous) and the number of bouts self-scratches occurring during the last sub-block (continuous). The log-transformed value of the duration of the current sub-block (in minutes) was used as the offset term. We addressed pseudoreplication by including subject ID as a random intercept. The significance of the full model was confirmed through comparison with a null model containing control variables and a random intercept, using a likelihood ratio test with the anova function (electronic supplementary material, table S2).

## 3. Results

**Figure 2.**
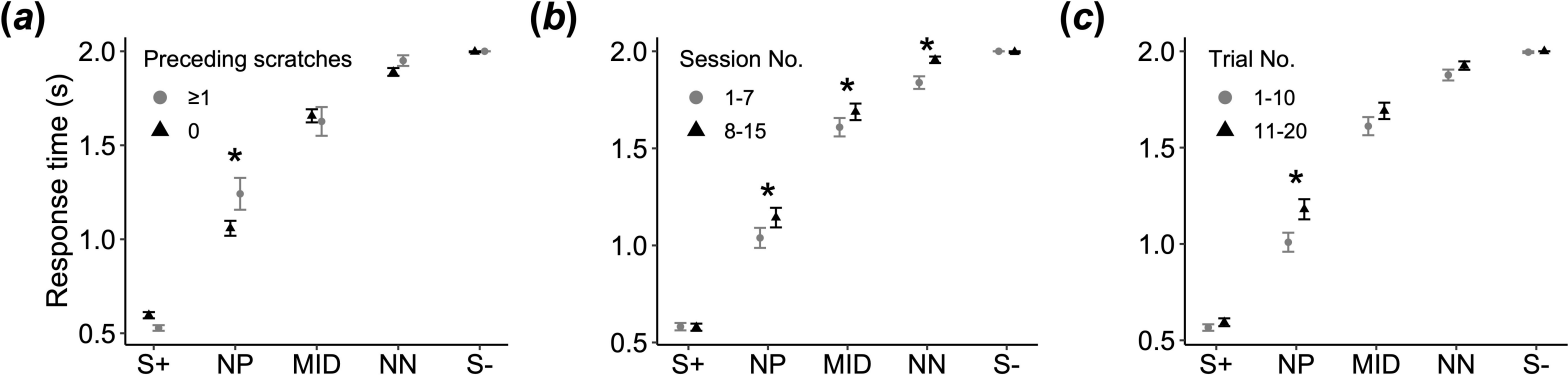
Factors affecting the response time in the judgement bias test. (*a*) The mean response time according to the number of self-scratching bouts during the period between the end of the last test trial and the beginning of the current test trial. For illustration purposes, we grouped the response times from cases where self-scratching occurred at least once during this period. Asterisks denote a significant interaction term between the number of self-scratching bouts and the type of test stimulus. (*b*) The mean response time according to the session number. We separated the response times from the first half of the sessions and the second half of the sessions. Asterisks denote a significant interaction term between the session number and the type of test stimulus. (*c*) The mean response time according to the trial number. For illustration purposes, we grouped the response times from the first 10 trials and the last 10 trials. Asterisks denote a significant interaction term between the trial number and the type of test stimulus. Error bars represent standard errors. *p < 0.05 (GLMM with a gamma error structure). Sample size: *n* = 1800.

Overall, our results are best consistent with the *BR→CM hypothesis*, demonstrating that the occurrence of self-scratching predicts a subsequent pessimistic judgement of ambiguous stimuli, while pessimistic judgements do not predict the subsequent occurrence of self-scratching. For the response time in the judgement bias test, the full model accounted for a significantly greater variance than the null model ($\chi_{53}^{2}$＝1657.3, *p* < 0.0001; electronic supplementary material, table S2). The results indicated that the response time to NP was significantly prolonged if the individuals were engaged in self-scratching during the period between the end of the last judgement bias test trial and the beginning of the current judgement bias test trial (GLMM: test stimulus (NP) × preceding scratching, *β* = 0.135 ± 0.061, *p* = 0.026; figure 2*a*; electronic supplementary material, table S3; consistent with prediction 1). The statistical model also revealed that the response times to NP, MID and NN increased with the session number (GLMM: test stimulus (NP) × session number, *β* = 0.014 ± 0.007, *p* = 0.041; test stimulus (MID) × session number, *β* = 0.014 ± 0.007, *p* = 0.029; test stimulus (NN) × session number, *β* = 0.014 ± 0.007, *p* = 0.031; figure 2*b*; electronic supplementary material, table S3), implying a diminished responsiveness to ambiguous stimuli owing to the individuals learning that these stimuli were non-rewarding. Response time to NP increased as the number of trials within each session increased, suggesting that learning about the unrewarding nature of an ambiguous stimulus might have occurred even within each session (GLMM: test stimulus (NP) × trial number, *β* = 0.010 ± 0.005, *p* = 0.041; figure 2*c*). The condition (video type) exerted no significant influence on the response time to the test stimuli.

**Figure 3.**
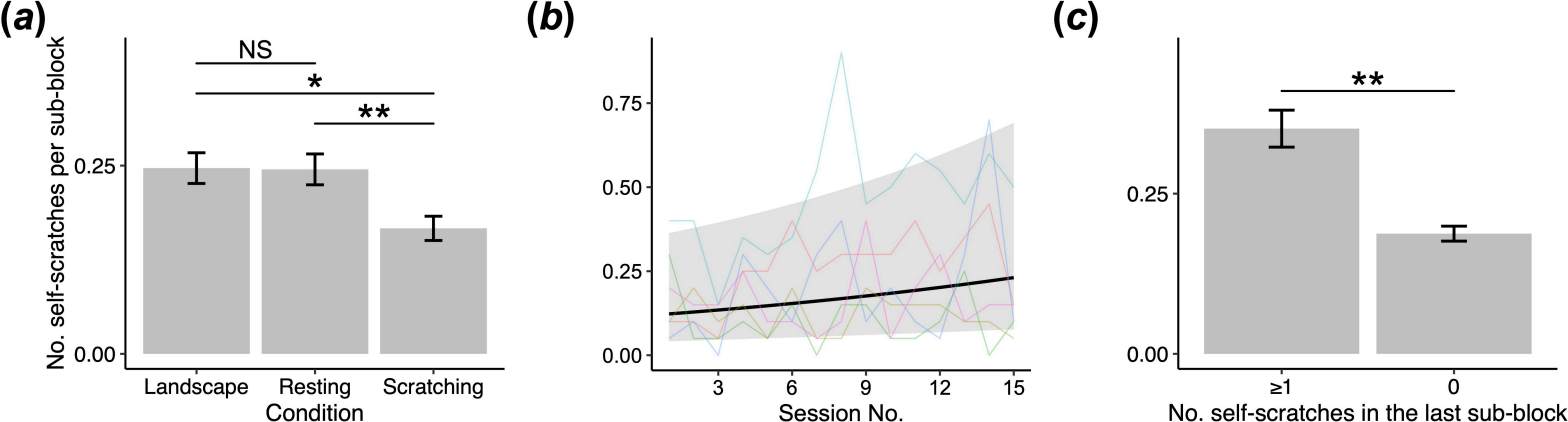
Factors affecting the occurrence of self-scratching in the sub-block between the end of the last judgement bias test trial and the onset of the current judgement bias test trial. (*a*) The mean number of self-scratching bouts according to the condition. **p < 0.01, *p < 0.05, NS: non-significant (post hoc Tukey’s tests). (*b*) The mean number of self-scratching bouts according to the session number. The black line and shaded area represent the fitted values and 95% confidence intervals, respectively. Coloured lines represent individual subjects. (*c*) The mean number of self-scratching bouts according to the number of self-scratching bouts in the last sub-block. For illustration purposes, we grouped the cases where self-scratching occurred at least once in the last sub-block. Asterisks denote a significant effect of the number of self-scratching bouts in the last sub-block. **p < 0.01 (GLMM with a Poisson error structure). Sample size: *n* = 1800.

Regarding the occurrence of self-scratching in the sub-block between the end of the last judgement bias test trial and the onset of the current judgement bias test trial, the full model accounted for a significantly greater variance than the null model ($\chi_{11}^{2}$= 23.9, *p* = 0.013; electronic supplementary material, table S2). Our results indicated that self-scratching was not influenced by the response time to the ambiguous stimuli (i.e. the degree of pessimistic judgement) in the last judgement bias test trial (GLMM: test stimulus (NP) × response time, *β* = 0.026 ± 0.543, *p* = 0.961; test stimulus (MID) × response time, *β* = −0.416 ± 0.560, *p* = 0.457; test stimulus (NN) × response time, *β* = −0.066 ± 0.720, *p* = 0.927; electronic supplementary material, table S4) (inconsistent with prediction 2). Unexpectedly, subjects were significantly less likely to scratch themselves in the scratching video condition compared with the resting video or landscape video conditions (post hoc Tukey’s test: landscape versus scratching, *β* = 0.352 ± 0.135, *p* = 0.025; resting versus scratching, *β* = 0.399 ± 0.131, *p* = 0.006; figure 3*a*; electronic supplementary material, table S5). No significant difference was observed in the likelihood of self-scratching between the resting video condition and the landscape condition (post hoc Tukey’s test: landscape versus resting, *β* = −0.046 ± 0.124, *p* = 0.930; figure 3*a*; electronic supplementary material, table S5). The likelihood of scratching increased with the session number (GLMM: session number, *β* = 0.032 ± 0.012, *p* = 0.010; figure 3*b*; electronic supplementary material, table S4). This could be attributed to the subjects learning about the unrewarding nature of ambiguous stimuli over the course of the sessions, thereby decreasing the expected value of reward per session. Additionally, subjects who had engaged in self-scratching in the last sub-block were significantly more likely to scratch again in the current sub-block than subjects who had not (GLMM: preceding scratching, *β* = 0.232 ± 0.090, *p* = 0.010; figure 3*c*; electronic supplementary material, table S4), suggesting scratching tends to occur in succession.

## 4. Discussion

Our findings suggest that the peripheral bodily responses (i.e. self-scratching) of Japanese macaques would predict a pessimistic cognitive judgement, but not the other way around. There was no tendency for response times to trained unambiguous stimuli to increase after self-scratching. Therefore, this indicates that self-scratching did not result in an overall decrease in reaction speed, but led to pessimistic judgements specifically in ambiguous situations (i.e. the subjects reduced their expectation of a positive outcome and/or increased their anticipation of an unfavourable outcome). Research exploring the relationship between bodily (e.g. behaviour, physiology) and cognitive (e.g. judgement, decision-making, reasoning) changes associated with affective states has been exclusively centred on humans, who possess the ability to reflect on and verbally report their own affective experiences [16,17]. Therefore, the findings of the current study are significant from a comparative perspective when considering the evolutionary foundations of the human affective response system. The present results can be construed as being in line with the classical facial expression feedback hypothesis [40], suggesting that the afferent pathway from bodily responses to alterations in cognitive judgements also exists in non-human primates (it should be noted, however, that subsequent studies have questioned the reproducibility of the facial expression feedback hypothesis [53,54]). Our findings may also be relevant to the propositions of the dual process theory of cognitive information processing [55]. This theory posits two processing systems: system 1 and system 2. System 1 is fast, automatic, subconscious and associated with immediate bodily reactions, while system 2 is slow, demands attention and effort, operates under conscious control and relates to subjective experience. Advocates of this theory maintain that, at least in humans, system 1 underpins system 2, with system 2 monitoring and, if necessary, modifying system 1 [55]. Considering our results in light of this theory, self-scratching could be associated with system 1, and changes in the mode of judgement could be linked to system 2. Our results suggest that in non-human primates, too, the rapid-acting system 1 (i.e. self-scratching) precedes and underlies the regulation of system 2 (i.e. the pessimistic mode of cognition). We speculate that these afferent pathways, demonstrated by our findings, may confer adaptive utility in animals’ natural habitats by enabling them to first rapidly respond to immediate demands at the bodily level, and then engage in more time-consuming cognitive information processing—including judgement—as part of their overall coping strategy.

Despite findings in humans suggesting that the pessimistic mode of thinking and cognition triggers efferent changes [56,57], such an influence was not observed in Japanese macaques in the current study. The precise reasons for this remain unclear based solely on this study. However, possible explanations may include the following: first, the pessimistic cognitive mode elicited in the subjects during the experimental session might have been so mild that it was inadequate to provoke additional physical changes. Given that the condition (i.e. type of video) had no significant impact on the judgement bias, it is plausible that the pessimistic cognitive mode induced in the subjects during the test session could have resulted from mild frustration owing to the insertion of the video during the task, regardless of its content, and was not accompanied by as strong a negative valence as, for example, the fear induced by stimuli related to predators [58]. Second, it is possible that the pessimistic cognitive mode might have indeed triggered peripheral bodily changes, yet these changes did not manifest as self-scratching. Although the shift in cognitive mode did not elicit the conspicuous bodily response of self-scratching, it is quite plausible that it did induce certain physiological responses, such as changes in heart rate or skin conductance. The third possibility, which is particularly intriguing from an evolutionary perspective, is that self-reflective cognition is essential for the pessimistic mode of cognition to recurrently induce bodily change. In other words, metacognitive awareness of one’s own pessimism might amplify the bodily responses associated with negative affect. Indeed, human studies have demonstrated that metacognition serves as a mediator between perceived stress and ensuing negative affect [59] and that awareness of one’s affective state accelerates autonomic vagal activity [41]. Considering that recent studies have suggested the presence of metacognitive abilities in non-human animals [60], it would be an intriguing topic for future research to explore whether qualitative differences in metacognition between humans and non-human animals influence the relationship between pessimistic cognitive modes and bodily responses.

Our results indicated that the response times were significantly extended only for NP following self-scratching, but not for MID or NN. Indeed, judgement bias does not always manifest most prominently with stimuli that lie precisely midway between S+ and S– in terms of physical values [48] (reviewed in [22]). NP might have represented the greatest ambiguity for our subjects, and consequently, the most pronounced judgement bias might have been observed with this stimulus. Furthermore, the strongest bias in judgement was observed with NP in a previous study that employed the same paradigm and species [46].

It is worth mentioning that the pessimistic judgement bias following self-scratching was robust enough that the confounding learning effect of repeated testing did not eliminate it. Several previous studies have warned that the impact of learning on animals’ responses to ambiguous stimuli may significantly confound the interpretation of results from judgement bias tests [48,61,62]. To mitigate the learning effect [47,48,63], we decreased the ratio of ambiguous stimulus trials relative to reference stimulus trials in each session (303 : 20) and applied an 80% variable reinforcement schedule. Despite these measures, the response times to all types of ambiguous stimuli slowed down over successive sessions, indicating that the subjects may have learned that ambiguous stimuli would not lead to rewards. Specifically, for NP, the response times increased as the trial count rose, even within individual sessions. To further attenuate learning effects, it may be necessary to significantly lower the ratio of ambiguous stimuli, implement a variable reinforcement schedule with lower percentages, or employ ambiguous and reference stimuli that are more difficult to differentiate.

Unexpectedly, despite numerous prior studies demonstrating that self-scratching is contagious in both humans and non-human animals (e.g. [32,64]), the contagion was not observed in the current study. There are several possible reasons for this result: first, the presentation of video-recorded scratching may have suppressed contagion. While some studies have demonstrated that video-recorded stimuli can also induce contagious scratching (humans: [65]; mice: [33]; rhesus macaques: [34]), several other studies indicate that video-recorded stimuli do not induce contagion like live conspecific stimuli (mice: [38]; Barbary macaques, *Macaca sylvanus*: [37]). Given the inconsistency in the results from previous studies, we cannot draw any definitive conclusions. However, it is plausible that some aspects of the video-recorded stimuli might have been unnatural to the subjects, thereby skewing their responses. Second, our video stimuli did not contain any audio. Previous studies in humans have shown that scratching sounds trigger contagious itch [66,67]. Scratching sounds may be a critical factor for the occurrence of contagion in non-human animals as well. Third, the individuals featured in the video stimuli were unfamiliar to our subjects. We used videos of unfamiliar individuals because the only study examining familiarity bias in scratching contagion in non-human animals has shown that this behaviour exhibits stronger contagion among weakly bonded individuals [35]. However, we cannot discount the possibility of interspecies differences in the influence of familiarity, and that contagion might occur in our subject species if we employ video stimuli of familiar individuals.

Several lingering questions remain that should be addressed by future research. First, while the current study suggested that peripheral behavioural responses associated with negative affect unidirectionally predict changes in cognitive mode, the causal mechanisms linking these aspects remain unclear. By investigating the causal mechanisms using neurological and physiological approaches, future studies will provide a more comprehensive understanding of the mechanisms underlying affective response systems. Second, while the James–Lange hypothesis (particularly William James’ series of studies) focused on subjective affective feelings [9], our data do not provide information about the subjective experience in non-human animals, but only about the mode of cognitive judgement associated with a specific affect. Although it may be possible to have animals report their own affective state of feeling by applying an experimental paradigm of metacognition (e.g. [68]) that quantifies an animal’s confidence level in a specific task, we must exercise caution when dealing with objective measures of subjective feeling in non-human animals [69].

## 5. Conclusions

The current study suggests that specific preceding peripheral bodily responses in Japanese macaques predict subsequent pessimistic judgement bias, but not vice versa. From an evolutionary standpoint, it is intriguing that this study did not identify any efferent pathways from cognitive changes to bodily responses. Given that metacognition modulates affective responses in humans, further investigation is needed to determine if self-reflective cognition similarly supports efferent pathways in non-human animals. Regardless of the underlying mechanism, the coping strategy of first addressing immediate needs through rapid-acting bodily responses and then engaging in cognitive information processing involving time-consuming judgements is probably adaptive for dealing with challenges in natural habitats and, therefore, may have been evolutionarily conserved.

## Data Availability

All data and R codes have been deposited on OSF [70]. Supplementary material is available online [71].

## References

[1] Dolan RJ. 2002 Emotion, cognition, and behavior. Science **298**, 1191–1194. (10.1126/science.1076358) 12424363

[2] Pessoa L. 2008 On the relationship between emotion and cognition. Nat. Rev. Neurosci. **9**, 148–158. (10.1038/nrn2317) 18209732

[3] Anderson DJ, Adolphs R. 2014 A framework for studying emotions across species. Cell **157**, 187–200. (10.1016/j.cell.2014.03.003) 24679535 PMC4098837

[4] Darwin C. 1872 The expression of the emotions in man and animals. London, UK: John Murray.

[5] James W. 1894 Discussion: the physical basis of emotion. Psychol. Rev. **1**, 516–529. (10.1037/h0065078) 8022955

[6] James W. 1884 What is an emotion? Mind **9**, 188–205. (10.1093/mind/os-IX.34.188)

[7] de Spinoza B. 1994 A Spinoza reader: the ethics and other works. Princeton, NJ: Princeton University Press.

[8] Lange CG, James W. 1922 The emotions. Baltimore, MD: Williams & Wilkins Co.

[9] Lang PJ. 1994 The varieties of emotional experience: a meditation on James-Lange theory. Psychol. Rev. **101**, 211–221. (10.1037/0033-295x.101.2.211) 8022956

[10] Friedman BH. 2010 Feelings and the body: the Jamesian perspective on autonomic specificity of emotion. Biol. Psychol. **84**, 383–393. (10.1016/j.biopsycho.2009.10.006) 19879320

[11] Schachter S, Singer J. 1962 Cognitive, social, and physiological determinants of emotional state. Psychol. Rev. **69**, 379–399. (10.1037/h0046234) 14497895

[12] Lazarus RS, Averill JR, Opton EM. 1970 Towards a cognitive theory of emotion. In Feelings and emotions (ed. MB Arnold), pp. 207–232. New York, NY: Academic Press. (10.1016/B978-0-12-063550-4.50023-1)

[13] Damasio A. 1994 Descartes’ error: emotion, reason, and the human brain. New York, NY: Putnam Pub Group.

[14] Damasio A. 2003 Looking for Spinoza: joy, sorrow, and the feeling brain. San Diego, CA: Harcourt.

[15] Prinz J. 2004 Gut reactions: a perceptual theory of emotion. Oxford, UK: Oxford University Press.

[16] Panksepp J. 2011 Toward a cross‐species neuroscientific understanding of the affective mind: do animals have emotional feelings? Am. J. Primatol. **73**, 545–561. (10.1002/ajp.20929) 21319205

[17] Panksepp J. 2012 What is an emotional feeling? Lessons about affective origins from cross-species neuroscience. Motiv. Emot. **36**, 4–15. (10.1007/s11031-011-9232-y)

[18] Bohn-Gettler CM, Rapp DN. 2011 Depending on my mood: mood-driven influences on text comprehension. J. Educ. Psychol. **103**, 562–577. (10.1037/a0023458) 21927504 PMC3172144

[19] Isen A. 2008 Some ways in which positive affect influences decision making and problem solving. In Handbook of emotions, 3rd edn (eds M Lewis, J Haviland-Jones, L Barrett), pp. 548–573. New York, NY: The Guilford Press.

[20] Paul ES, Harding EJ, Mendl M. 2005 Measuring emotional processes in animals: the utility of a cognitive approach. Neurosci. Biobehav. Rev. **29**, 469–491. (10.1016/j.neubiorev.2005.01.002) 15820551

[21] Mendl M, Burman OHP, Parker RMA, Paul ES. 2009 Cognitive bias as an indicator of animal emotion and welfare: emerging evidence and underlying mechanisms. Appl. Anim. Behav. Sci. **118**, 161–181. (10.1016/j.applanim.2009.02.023)

[22] Lagisz M, Zidar J, Nakagawa S, Neville V, Sorato E, Paul ES, Bateson M, Mendl M, Løvlie H. 2020 Optimism, pessimism and judgement bias in animals: a systematic review and meta-analysis. Neurosci. Biobehav. Rev. **118**, 3–17. (10.1016/j.neubiorev.2020.07.012) 32682742

[23] Neville V, Nakagawa S, Zidar J, Paul ES, Lagisz M, Bateson M, Løvlie H, Mendl M. 2020 Pharmacological manipulations of judgement bias: a systematic review and meta-analysis. Neurosci. Biobehav. Rev. **108**, 269–286. (10.1016/j.neubiorev.2019.11.008) 31747552 PMC6966323

[24] Schino G, Troisi A, Perretta G, Monaco V. 1991 Measuring anxiety in nonhuman primates: effect of lorazepam on macaque scratching. Pharmacol. Biochem. Behav. **38**, 889–891. (10.1016/0091-3057(91)90258-4) 1871201

[25] Schino G, Perretta G, Taglioni AM, Monaco V, Troisi A. 1996 Primate displacement activities as an ethopharmacological model of anxiety. Anxiety **2**, 186–191. (10.1002/(sici)1522-7154(1996)2:43.0.co;2-m) 9160621

[26] Schino G, Rosati L, Geminiani S, Aureli F. 2007 Post‐conflict anxiety in Japanese macaques (Macaca fuscata): aggressor’s and victim’s perspectives. Ethology **113**, 1081–1088. (10.1111/j.1439-0310.2007.01407.x)

[27] Majolo B, Ventura R, Koyama NF. 2009 Anxiety level predicts post‐conflict behaviour in wild Japanese macaques (Macaca fuscata yakui). Ethology **115**, 986–995. (10.1111/j.1439-0310.2009.01685.x)

[28] Ueno M, Yamada K, Nakamichi M. 2015 Emotional states after grooming interactions in Japanese macaques (Macaca fuscata). J. Comp. Psychol. **129**, 394–401. (10.1037/a0039688) 26348969

[29] Castles DL, Whiten A. 1998 Post‐conflict behaviour of wild olive baboons. II. stress and self‐directed behaviour. Ethology **104**, 148–160. (10.1111/j.1439-0310.1998.tb00058.x)

[30] Kutsukake N. 2003 Assessing relationship quality and social anxiety among wild chimpanzees using self-directed behaviour. Behaviour **140**, 1153–1171. (10.1163/156853903322589687)

[31] Palagi E, Norscia I. 2011 Scratching around stress: hierarchy and reconciliation make the difference in wild brown lemurs (Eulemur fulvus). Stress **14**, 93–97. (10.3109/10253890.2010.505272) 20666657

[32] Papoiu ADP, Wang H, Coghill RC, Chan YH, Yosipovitch G. 2011 Contagious itch in humans: a study of visual ‘transmission’ of itch in atopic dermatitis and healthy subjects. Br. J. Dermatol. **164**, 1299–1303. (10.1111/j.1365-2133.2011.10318.x) 21410682 PMC3110738

[33] Yu YQ, Barry DM, Hao Y, Liu XT, Chen ZF. 2017 Molecular and neural basis of contagious itch behavior in mice. Science **355**, 1072–1076. (10.1126/science.aak9748) 28280205 PMC5502115

[34] Feneran AN *et al*. 2013 Monkey see, monkey do: contagious itch in nonhuman primates. Acta Derm. Venereol. **93**, 27–29. (10.2340/00015555-1406) 22735614

[35] Laméris DW, van Berlo E, Sterck EHM, Bionda T, Kret ME. 2020 Low relationship quality predicts scratch contagion during tense situations in orangutans (Pongo pygmaeus). Am. J. Primatol. **82**, e23138. (10.1002/ajp.23138) 32333423 PMC7379188

[36] Valdivieso-Cortadella S, Bernardi-Gómez C, Aureli F, Llorente M, Amici F. 2023 Yawning and scratching contagion in wild spider monkeys (Ateles geoffroyi). Sci. Rep. **13**, 8367. (10.1038/s41598-023-35693-5) 37225745 PMC10209189

[37] Whitehouse J, Micheletta J, Kaminski J, Waller B. 2016 Macaques attend to scratching in others. Anim. Behav. **122**, 169–175. (10.1016/j.anbehav.2016.10.020)

[38] Lu JS, Chen QY, Zhou SB, Wu FY, Liu RH, Zhou ZX, Zhang H, Zhuo M. 2019 Contagious itch can be induced in humans but not in rodents. Mol. Brain **12**, 38. (10.1186/s13041-019-0455-2) 31014383 PMC6480616

[39] Strack F, Martin L, Stepper S. 1988 Inhibiting and facilitating conditions of the human smile: a nonobtrusive test of the facial feedback hypothesis. J. Personal. Soc. Psychol. **54**, 768–777. (10.1037/0022-3514.54.5.768) 3379579

[40] Zajonc RB, Murphy ST, Inglehart M. 1989 Feeling and facial efference: implications of the vascular theory of emotion. Psychol. Rev. **96**, 395–416. (10.1037/0033-295x.96.3.395) 2756066

[41] Kanbara K, Fukunaga M. 2016 Links among emotional awareness, somatic awareness and autonomic homeostatic processing. Biopsychosoc. Med. **10**, 16. (10.1186/s13030-016-0059-3) 27175214 PMC4863353

[42] Palagi E, Norscia I, Demuru E. 2014 Yawn contagion in humans and bonobos: emotional affinity matters more than species. PeerJ **2**, e519. (10.7717/peerj.519) 25165630 PMC4137654

[43] Romero T, Konno A, Hasegawa T. 2013 Familiarity bias and physiological responses in contagious yawning by dogs support link to empathy. PLoS ONE **8**, e71365. (10.1371/journal.pone.0071365) 23951146 PMC3737103

[44] Romero T, Ito M, Saito A, Hasegawa T. 2014 Social modulation of contagious yawning in wolves. PLoS ONE **9**, e105963. (10.1371/journal.pone.0105963) 25162677 PMC4146576

[45] Gallo A, Zanoli A, Caselli M, Palagi E, Norscia I. 2021 First evidence of yawn contagion in a wild monkey species. Sci. Rep. **11**, 17957. (10.1038/s41598-021-96423-3) 34504125 PMC8429631

[46] Iki S, Adachi I. 2023 Fearful snake pictures make monkeys pessimistic. iScience **26**, 107622. (10.1016/j.isci.2023.107622) 37664603 PMC10474457

[47] Bethell E, Holmes A, MacLarnon A, Semple S. 2012 Cognitive bias in a non-human primate: husbandry procedures influence cognitive indicators of psychological well-being in captive rhesus macaques. Anim. Welf. **21**, 185–195. (10.7120/09627286.21.2.185)

[48] Bethell EJ, Koyama NF. 2015 Happy hamsters? Enrichment induces positive judgement bias for mildly (but not truly) ambiguous cues to reward and punishment in Mesocricetus auratus. R. Soc. Open Sci. **2**, 140399. (10.1098/rsos.140399) 26587255 PMC4632568

[49] Crump A, Jenkins K, Bethell EJ, Ferris CP, Kabboush H, Weller J, Arnott G. 2021 Optimism and pasture access in dairy cows. Sci. Rep. **11**, 4882. (10.1038/s41598-021-84371-x) 33649476 PMC7921385

[50] McCoy DE, Schiestl M, Neilands P, Hassall R, Gray RD, Taylor AH. 2019 New Caledonian crows behave optimistically after using tools. Curr. Biol. **29**, 2737–2742.(10.1016/j.cub.2019.06.080) 31378612

[51] Mundry R, Nunn CL. 2009 Stepwise model fitting and statistical inference: turning noise into signal pollution. Am. Nat. **173**, 119–123. (10.1086/593303) 19049440

[52] Harrell FE. 2015 Multivariable modeling strategies. In Regression modeling strategies. Springer series in statistics, pp. 63–102. Cham, Switzerland: Springer. (10.1007/978-3-319-19425-7_4)

[53] Coles NA *et al*. 2022 A multi-lab test of the facial feedback hypothesis by the Many Smiles Collaboration. Nat. Hum. Behav. **6**, 1731–1742. (10.1038/s41562-022-01458-9) 36266452

[54] Acosta A *et al*. 2016 Registered replication report: Strack, Martin, & Stepper (1988). Perspect. Psychol. Sci. **11**, 917–928. (10.1177/1745691616674458) 27784749

[55] Kahneman D. 2011 Thinking, fast and slow. New York, NY: Farrar, Straus and Giroux.

[56] Thayer JF, Friedman BH, Borkovec TD. 1996 Autonomic characteristics of generalized anxiety disorder and worry. Biol. Psychiatry **39**, 255–266. (10.1016/0006-3223(95)00136-0) 8645772

[57] Hofmann SG, Moscovitch DA, Litz BT, Kim HJ, Davis LL, Pizzagalli DA. 2005 The worried mind: autonomic and prefrontal activation during worrying. Emotion **5**, 464–475. (10.1037/1528-3542.5.4.464) 16366750

[58] Öhman A, Mineka S. 2003 The malicious serpent: snakes as a prototypical stimulus for an evolved module of fear. Curr. Dir. Psychol. Sci. **12**, 5–9. (10.1111/1467-8721.01211)

[59] Spada M, Nikčević A, Moneta G, Wells A. 2008 Metacognition, perceived stress, and negative emotion. Personal. Individ. Differ. **44**, 1172–1181. (10.1016/j.paid.2007.11.010)

[60] Hampton R. 2019 Monkey metacognition could generate more insight. Anim. Behav. Cogn. **6**, 230–235. (10.26451/abc.06.04.02.2019) 33834091 PMC8025984

[61] Doyle RE, Vidal S, Hinch GN, Fisher AD, Boissy A, Lee C. 2010 The effect of repeated testing on judgement biases in sheep. Behav. Process. **83**, 349–352. (10.1016/j.beproc.2010.01.019) 20117188

[62] Wilson C, Hall N, Aviles-Rosa EO, Campbell K, Arnott G, Reeve C. 2023 The effect of repeated testing on judgement bias in domestic dogs (Canis familiaris). Anim. Cogn. **26**, 477–489. (10.1007/s10071-022-01689-3) 36094748 PMC9465138

[63] Hintze S, Melotti L, Colosio S, Bailoo JD, Boada-Saña M, Würbel H, Murphy E. 2018 A cross-species judgement bias task: integrating active trial initiation into a spatial go/no-go task. Sci. Rep. **8**, 5104. (10.1038/s41598-018-23459-3) 29572529 PMC5865189

[64] Nakayama K. 2004 Observing conspecifics scratching induces a contagion of scratching in Japanese monkeys (Macaca fuscata). J. Comp. Psychol. **118**, 20–24. (10.1037/0735-7036.118.1.20) 15008669

[65] Holle H, Warne K, Seth AK, Critchley HD, Ward J. 2012 Neural basis of contagious itch and why some people are more prone to it. Proc. Natl Acad. Sci. USA **109**, 19816–19821. (10.1073/pnas.1216160109) 23150550 PMC3511754

[66] Swithenbank S, Cowdell F, Holle H. 2016 The role of auditory itch contagion in psoriasis. Acta Derm. Venereol. **96**, 728–731. (10.2340/00015555-2320) 26676729

[67] Meeuwis SH, Skvortsova A, van Laarhoven AIM, Holle H, Evers AWM. 2022 Can contagious itch be affected by positive and negative suggestions? Exp. Dermatol. **31**, 1853–1862. (10.1111/exd.14663) 36048562 PMC10087404

[68] Smith JD, Schull J, Strote J, McGee K, Egnor R, Erb L. 1995 The uncertain response in the bottlenosed dolphin (Tursiops truncatus). J. Exp. Psychol. Gen. **124**, 391–408. (10.1037/0096-3445.124.4.391) 8530911

[69] Hampton RR. 2009 Multiple demonstrations of metacognition in nonhumans: converging evidence or multiple mechanisms? Comp. Cogn. Behav. Rev. **4**, 17–28. (10.3819/ccbr.2009.40002) 20046911 PMC2748335

[70] Iki S, Adachi I. 2025 Data for: Affective bodily responses in monkeys predict subsequent pessimism, but not vice versa. OSF. See https://osf.io/nvk7s/.10.1098/rspb.2024.254939904392

[71] Iki S, Adachi I. 2025 Supplementary material from: Affective bodily responses in monkeys predict subsequent pessimism, but not vice versa. Figshare (10.6084/m9.figshare.c.7652248)39904392

